# Reduction of Photo Bleaching and Long Term Archiving of Chemically Cleared GFP-Expressing Mouse Brains

**DOI:** 10.1371/journal.pone.0114149

**Published:** 2014-12-02

**Authors:** Klaus Becker, Christian Markus Hahn, Saiedeh Saghafi, Nina Jährling, Martina Wanis, Hans-Ulrich Dodt

**Affiliations:** 1 Vienna University of Technology, FKE, Chair of Bioelectronics, Vienna, Austria; 2 Center for Brain Research, Medical University of Vienna, Vienna, Austria; Charité-Universitätsmedizin Berlin, Germany

## Abstract

Tissue clearing allows microscopy of large specimens as whole mouse brains or embryos. However, lipophilic tissue clearing agents as dibenzyl ether limit storage time of GFP-expressing samples to several days and do not prevent them from photobleaching during microscopy. To preserve GFP fluorescence, we developed a transparent solid resin formulation, which maintains the specimens' transparency and provides a constant signal to noise ratio even after hours of continuous laser irradiation. If required, high-power illumination or long exposure times can be applied with virtually no loss in signal quality and samples can be archived for years.

## Introduction

Tissue clearing using various chemicals allows visualizing the inside of large specimens as entire mouse brains or mouse embryos with modern 3D-microscopy, as ultramicroscopy. Most of these clearing techniques rely on replacing the water content of a sample by a liquid matching the refractive index of proteins [1]. Thus light scattering is reduced and specimens appear translucent if light absorption within the tissue is negligible [1], [2].

A widely used chemical tissue clearing reagent is Murray's clearing medium (BABB) composed of one volume part benzyl alcohol (BA), and two volume parts benzyl benzoate (BB) [3]. As recently shown, dibenzyl ether (DBE) is a clearing medium that provides improved tissue transparency and fluorescence preservation compared to BABB [4]. However, neither BABB nor DBE prevent fluorescent samples from photobleaching due to massive light exposure, as required by certain microscopy techniques as STED microscopy [5]. Since, there is also some light-independent fluorescence decline, both clearing agents are not suitable for long-term storage of GFP-expressing specimens beyond a few days.

Here, we introduce a resin, which efficiently protects cleared samples from fluorescence fading, photobleaching and mechanical damage without impairing their transparency.

## Materials and Methods

### Preparation of cleared mouse brains or hippocampi

Formaldehyde-fixed brains obtained from transcardially perfused Thy-1 GFP-M (C57/Bl6) mice [6] were split into hemispheres, dehydrated and cleared with tetrahydrofuran and DBE as described in [4]. If required, hippocampi were dissected directly after fixation. Animal care and euthanasia was done in accordance with the ethics guidelines of the Austrian animal protection law. The animals were sacrificed by the authors of this study using carbon dioxide at the Center for Brain Research prior to any experimental manipulation. Death was confirmed by lack of respirational movements for at least 1 min and any movement reaction on firm toe pinching. According to §2 of the Austrian animal experiments act, special approval by an ethics committee or an approval number was not required, since all experiments were not performed on living animals. Breeding and raising of the mice was done at the Animal Facility of the Medical University of Vienna.

### Fabrication of embedding molds

**Figure 1:**
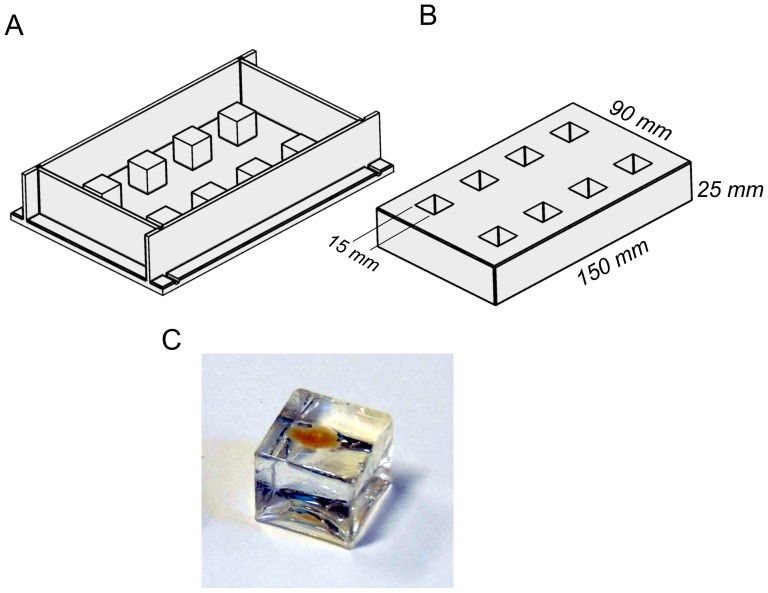
Fabrication of molds for resin embedding from Silastic E-RTV silicone rubber. (**A**) Casting frame made from acrylic glass. (**B**) Silicon rubber mold. (**C**) Cured resin block with embedded cleared mouse brain hemisphere.

Resin embedding was done in cubic silicon rubber molds of 15 mm side length for mouse brains and 10 mm side length for mouse hippocampi. The molds were fabricated from Silastic E-RTV silicone rubber (Dow Corning, Germany) according to [7]. Instead of using aluminum, we made the casting frames from acrylic glass providing a clean surface (Acrylic Glass GS, Senova GmbH, Austria) (**figure 1**). Curing of the molds was done for about 2h at 80°C. During the next day the cooled down molds were released from the frames.

### Measuring of refractive indices

Refractive indices of resin blocks were measured using an Abbe refractometer (Optech RMI, Carl Roth, Germany) at room temperature at daylight conditions. As a contact liquid between the resin block and the refractometer prism we used silicon oil (*n *> 1.7) (Type 705, Kurt Lesker GmbH, Germany).

### Preparation of the resin and resin embedding of the specimens

11.5 ml (at 50°C) of the epoxy resin D.E.R. 332 (bisphenol-A diglycidyl ether, Sigma-Aldrich Austria 31185), 3.5 ml of the flexibilizer D.E.R. 736 (polypropylene glycol diglycidyl ether, Sigma-Aldrich Austria 31191), and 3 ml of the cyclic allopathic amine epoxy curing reagent isophorone diamine (IPDA, 5-Amino-1,3,3-trimethylcyclohexanemethylamine, Sigma-Aldrich Austria 118184) were carefully mixed in a 50 ml falcon tube. Since D.E.R. 332 crystalizes slowly at room temperature, it was liquefied at 50°C in a water bath before use. The mixture was carefully mixed and degased in a vacuum chamber at about 100 mbar for about 60 minutes. This is essential to achieve homogeneous optical properties. Since IPDA is sensitive to oxygen, it should be stored under an inert gas as argon.

The specimens were pre-incubated in a small volume of resin mixture for about 15 min. Clearing solution was removed from the specimen's surface before by gently dabbing on a sheet of tissue paper. We filled the molds to about one third with the degassed resin mixture and embedded the specimens. Then the molds were completely filled up with resin mixture. Orientation of the specimens was adjusted by using an injection cannula. The resin cubes were cured at room temperature for at least 2 days in the dark. The cubes were fixed on small metal plates using silicone glue to prevent them from floating inside the DBE container during ultramicroscopy.

To find the resin formulation described above, several acrylic-, polyester- and epoxy-based resins were screened for their ability to preserve tissue transparency and fluorescence of cleared samples. Among them were the UV-curing acrylic resin Plexit 55 (Carl Roth, Germany) the polyester-based resins “Polyester-Klarharz” (Carl Roth, Germany) and “XOR crystal resin” (Hobbytime, Germany), and besides D.E.R. 332 and D.E.R. 736 the epoxy-based resins Araldite 506 (Sigma Aldrich, Austria), Biodur E12 and E13 (Biodur, Germany) and Epofix (Struers, Germany). Each epoxy resin was cured with different amine-based curing agents to find an optimal combination regarding tissue transparency and fluorescence. **Table 1** provides an overview of all tested combinations of resins and curing compounds.

**Table 1:** Tested resin compounds and curing agents.

	Tested resin compound	Tested curing agent	Remarks
Acrylic resins	Plexit 55 (Carl Roth, Germany)	UV light + catalyzer	Specimens become opaque
Epoxy resins	A. poly(bisphenol-A-co-epichlorhydrin) (Araldite 506), CAS 25068-38-6	**a**. 1,8-diamino-p-menthane, CAS 80-52-4	Intense background fluorescence in all tested combinations with A
	B. bisphenol A diglycidyl ether (D.E.R 332), CAS 1675-54-3	**b**. 2,2′-dimethyl-4,4′methylene-bis-(cylcohexylamine), CAS 6864-37-5	Differently pronounced loss of tissue transparency in all tested combinations of epoxy-resins (A-G) and curing agents (a-k) except composition D-e
	C. polypropylen glycol diglycidyl ether (D.E.R. 736), CAS 9072-62-2	**c**. 3-(aminomethyl)-benzylamin, CAS 246258-97-9	Combination D-e provides highest transparency and best GFP preservation
	D. Mixtures of bisphenol-A-diglycidyl ether (CAS 1675-54-3) and polypropylene glycol diglycidyl ether (CAS 9072-62-2)	**d**. 4,4′-diaminodiphenylmethane, CAS 101-77-9	
	E. E12	**e**. 5-amino-1,3,3-trimethyl-cyclo-hexane-methylamine (isophorone diamine), CAS 2855-13-2	
	F. E13	**f**. bis-(4-aminocyclohexyl)-methane, CAS 1761-71-3	
	G. Epofix	**g**. diethylene triamine, CAS 111-40-0	
	A-D obtained from Sigma-Aldrich Austria, E-F Biodur Germany, G Struers GmbH Germany	**h**. ethylendiamine, CAS 107-15-3	
		**i**. N-(2-aminoethyl) piperazine, CAS 140-31-8	
		**j**. m-xylenediamine, CAS 153326-45-5	
		**k**. triethylene tetramine, CAS 112-24-3	
		**l**. E1 curing agent.	
		**a-k** obtained from Sigma-Aldrich Austria, **l** from Biodur GmbH Germany	
Polyester resins	A. Polyester-Klarharz	a. methyl-ethyl-ketone peroxide (MEKP) curing agent	Formation of cracks in the resin at the surface of the specimens in all combinations with polyester resin
	B. Polyester-Klarharz with dibutyl phthalate as flexibilizer		Clouding of resin, if dibutyl- phthalate was added as a flexibilizer
	C. XOR crystal resin	**b**. lauroyl peroxide, CAS 105-74-8	Cleared specimens become opaque after a few days
	A-B obtained from Carl Roth Germany and Sigma-Aldrich Germany, C Hobbytime Germany	a obtained from Carl-Roth Germany, b from Sigma-Aldrich Austria	

The table gives an overview on the resins and curing compounds tested to achieve a transparent solid resin block preserving transparency and fluorescence of cleared samples.

### Ultramicroscopy

Imaging was performed using the ultramicroscopy setup described in [2]. During recording, the resin blocks were submerged in a DBE-filled container. Single-sided fluorescence excitation was performed by a 488 nm Sapphire laser (Coherent, Germany). All microscopy was done using a 4× Olympus XL Fluor objective (N.A. 0.28), which has been adapted for the refractive index of DBE by correction optics developed in our laboratory.

### Measurement and quantification of fluorescence intensities

**Figure 2:**
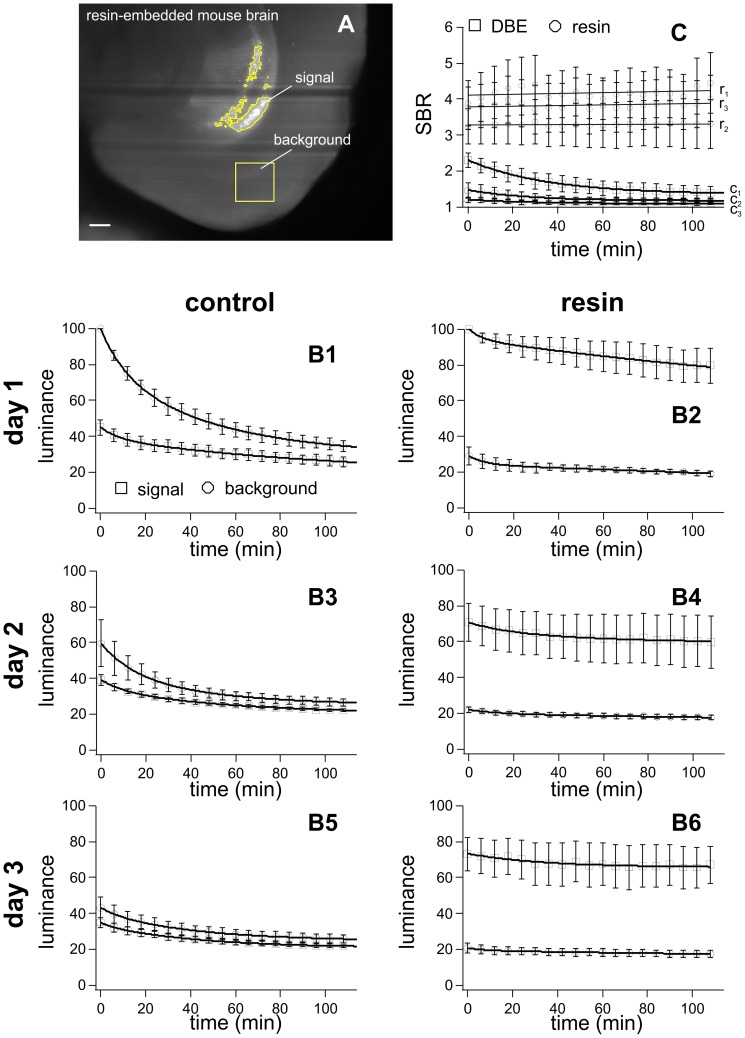
Quantification of fluorescence preservation during repetitive long-term illumination. Experiments were performed on three control and three resin-embedded samples. Each sample was exposed for 120 min on 3 consecutive days. Images were recorded any 6 min. On these images, mean pixel intensities were calculated in ROIs either covering areas exclusively containing fluorescence signal or background fluorescence. Size and location of the ROIs, were defined using the first image of each illumination interval. Length of scale bar: 300 µm. (**B1–B6**) Plots of the relative fluorescence intensity over time. Change of signal and background fluorescence intensities during three successive light exposures (day1 – day3) of 120 min duration each. Photobleaching is much lower in the resin-embedded sample than in the control and limited to the initial illumination phase. During light exposure signal to background ratios (SBRs  =  mean signal intensity/mean background intensity) remain constant for the resin embedded samples (r_1_, r_2_, r_3_), while they exponentially decrease in the controls (c_1_, c_2_, c_3_). The error bars indicate the standard error of the mean (SEM).

Using ultramicroscopy, corresponding thin layers inside two mouse brains and one mouse hippocampus (one of each resin-embedded and the other one in DBE as control) were illuminated on 3 consecutive days constantly for 2h at 30 mW. Images were recorded every 6 min using a CCD-camera (CoolSnap cf, Roper Scientific, Germany). Both, focus of the microscope and orientation of the specimens were kept constant. To quantify the resin-mediated fluorescence protection, we compared the fluorescence intensity within two corresponding regions of interest (ROI) in each resin-embedded and control sample. One of the ROIs contained signal and the other one contained exclusively background fluorescence (**figure 2A**). Sizes and locations of the ROIs were defined from the first image and kept the same for all images in the stacks. Calculations of average signal intensities and signal to background ratios (SBR  =  mean signal intensity/mean background intensity) were performed using ImageJ (National Institute of Health, USA) and Igor Pro 6.2 (Wavemetrics Inc., USA).

### Long- term storage of resin blocks

For archival storage, liquid was gently removed from the resin blocks using tissue paper. Then they were stored in small polystyrol boxes at room temperature in the dark.

## Results

**Figure 3:**
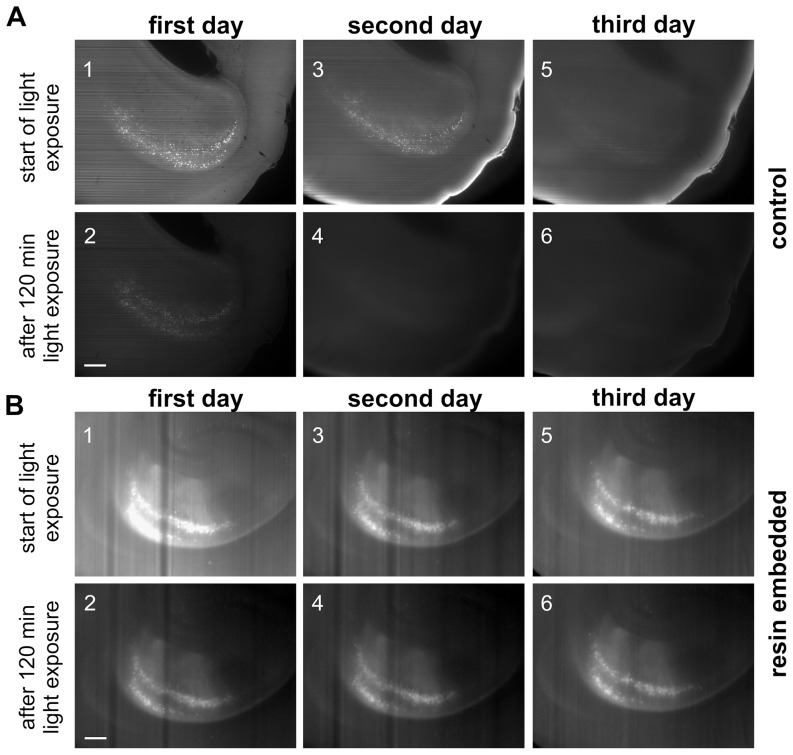
GFP signal preservation in resin-embedded mouse brain hemispheres. UM-images of a selected plane within the hippocampal area in both, a control (**A**) and a resin-embedded brain hemisphere (**B**), each recorded at onset (**1**, **3**, **5**) and after 120 min of constant high power illumination (**2**, **4**, **6**). (**A**) The control hemisphere shows a pronounced quenching of GFP-fluorescence during the illumination intervals. (**B**) In the resin-embedded hemisphere photobleaching is markedly reduced. Although a certain amount of photobleaching occurs during the first illumination interval (B2 vs. B1), the fluorescence intensity remains approximately stable during further light exposures. Note the recovery of fluorescence between illuminations. Length of scale bars: 150 µm.

During illumination, GFP exhibits a decay in fluorescence activity. Figure 3A shows photobleaching within a thin layer inside a cleared whole mouse brain hemisphere incubated in DBE. An optical section through the hippocampal region was exposed for 2h constantly to a high-power light sheet generated by an ultramicroscope. Illumination of the same layer was repeated on the following 2 days. During the first illumination period, the fluorescence dropped severely (**figure 3A**, left column). Overnight, fluorescence partially recovered in the dark. However, after the second illumination period, the signal was lost and did not recover (**figure 3A**). Under the same experimental conditions, fluorescence could be preserved largely by resin-embedding. After 2h constant illumination of a corresponding optical section of the resin-embedded hemisphere, considerably more fluorescence was present (**figure 3B**). Also inside resin, fluorescence recovered in between the illumination periods overnight in the dark. In contrast to the control, the recovery was almost complete and repeatable (**figure 3B**, third day**).** Concurrently with the mild fluorescence decline during illumination also autofluorescence was reduced and apparently fluorescence signal quality was maintained to a certain degree.

To quantify the resin-mediated fluorescence protection and signal maintenance, the fluorescence intensity within corresponding ROIs was analyzed (**figure 2A**). To this purpose three resin-embedded and three control samples were illuminated as described above and images were taken every 6min (**figure 2B**). From the average fluorescence intensities, signal to background ratios (SBR) were calculated (**figure 2C**). The SBR measures how much a fluorescent signal contrasts from the fluorescence of the background. In contrast to the SBRs of the control mouse brain hemispheres (**figure 2C**, c_1_–c_3_), the SBRs of the resin-embedded specimens (**figure 2C**, r_1_–r_3_) remained stable during the entire experiment, providing a constant and excellent signal quality. Loss in absolute signal brightness can be compensated by increasing the illumination intensity since the SBR is independent from the illumination intensity [8]. Contrarily, an exponential decay of SBR is observed in the control specimens (**figure 2C** c_1_) making the signal indistinguishable from the background after a few hours of illumination (**figure 2** **B3 and B5,** **figure 3A**).

**Figure 4:**
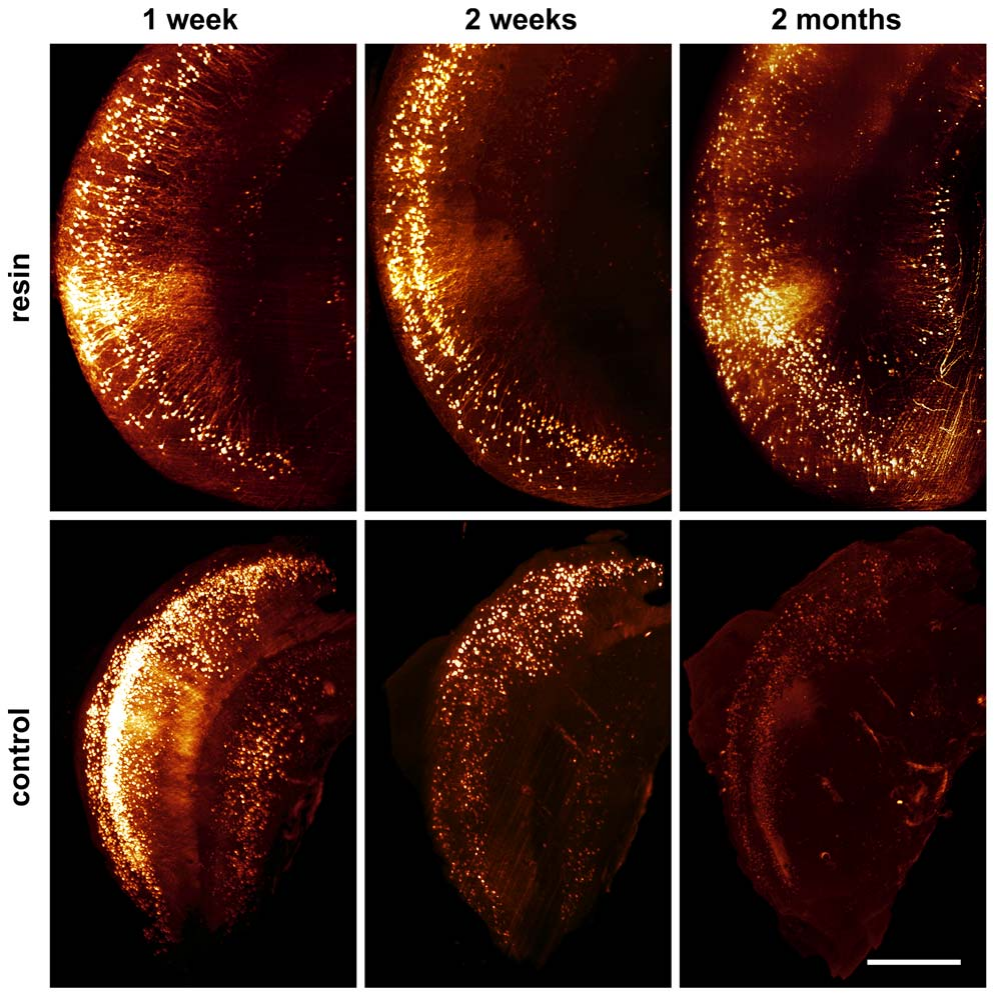
Fluorescence preservation by resin embedding during long-term storage. No loss of GFP signal quality in a resin-embedded mouse hippocampus can be observed within two months. In contrast, a hippocampus stored in DBE loses its fluorescence within less than two weeks. Length of scale bar: 150 µm.

Due to the fluorescence stabilizing effect of resin embedding, long-term storage of cleared GFP-expressing samples becomes possible e.g. for archiving purposes, or repetitive investigations. An embedded mouse hippocampus remains fluorescent over several months, without relevant loss in signal quality. Even after more than two years, a resin-embedded hippocampus was still fluorescent. In contrast, without embedding control samples completely lose fluorescence within a few days (**figure 4**).

To develop the resin formulation described here, various resins and curing compounds were screened. Table 1 provides an overview of the evaluated agents. The tested UV-curing acrylic resin Plexit 55 rendered samples opaque, presumably due to its low refractive index. The polyester resins “Polyester-Klarharz” and “XOR crystal resin” showed higher refractive indices > 1.56. However, embedded samples lost transparency within a few days and cracks developed at the surface between specimen and resin, probably due to the shrinkage of the polyester resins during curing. Addition of dibutyl phthalate as a flexibilizer reduced the shrinking artefacts, but induced light scattering. We tested the epoxy resins Araldite 506, Biodur E12, Biodur E13 and Epofix, but these resins affected tissue transparency severely. Finally we chose D.E.R. 332 as the basic embedding resin.

**Figure 5:**
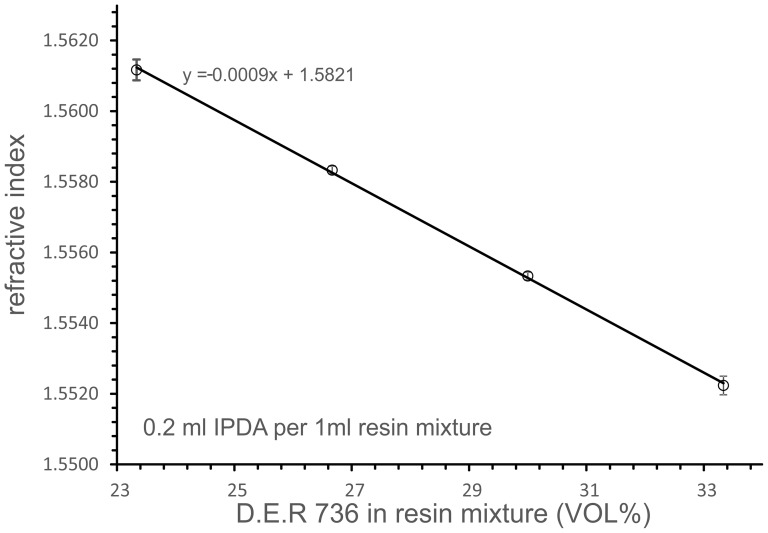
The refractive index of the cured resin depends on the relative amount of D.E.R.736 in the resin compound mixture (D.E.R.736 + D.E.R.332). By varying the percentage of D.E.R.736 the refractive index can be adjusted to different clearing reagents as DBE or BABB. The curing agent IPDA should be added 1:5 (vol/vol) to the premixed resin compound mixture.

The high refractive index of resin blocks made from D.E.R. 332 can be matched exactly to the refractive index of DBE by adding fractions of the lower refractive D.E.R. 736 as a second resin component, without affecting the quality of the resin block. A mixture of e.g. 3.85 ml D.E.R.736, 11.15 ml D.E.R.332 and 3 ml IPDA provides a resin block of *n* = 1.559, which equals the refractive index of BABB. The quantitative relation between the fraction of D.E.R. 736 in the resin compound mixture (i.e. D.E.R 332 and D.E.R 736, exclusively curing agent) and the refractive index of the cured resin is quantified in **figure 5**. D.E.R. 736 further acts as a flexibilizer preventing the cured resin blocks from becoming brittle.

## Discussion

We introduced a resin formulation that protects samples, which are cleared with DBE from fluorescence fading caused by high-energy illumination and long-term storage. The refractive index of the cured resin matches the refractive index of DBE (*n* = 1.561). Therefore light can pass without refraction from DBE to the resin and the resin-embedded specimen.

In DBE, peroxides and aldehydes are generated continuously in the presence of oxygen or water. This process is enhanced by light [9]. Peroxides can bind covalently to GFP quenching fluorescence permanently [10]. Resin-embedding probably prevents access of these molecules. Resin formulations resembling the one described in this paper are used in electron microscopy [11]–[13] and have also been applied to fluorescence light microscopy before [14]–. However, due to their high curing temperature and inappropriate refractive index they turned out to be unfeasible for preserving the transparency of cleared samples.

Therefore, we tested different resins and curing agents for their use as an embedding medium for cleared GFP-expressing biological tissues. Among these various combinations one composition provided best transparency and fluorescence preservation (**table 1**). Other compositions rendered the samples either opaque, caused severe autofluorescence, or did not cure under physiological temperature and pressure conditions. Tested polyester resins generally tend to form cracks near the surface between specimen and surrounding resin. Compared to polyester resins epoxy-based resins generally exhibit nearly no shrinkage during curing [17]. UV-curing methacrylates also provide low shrinkage, however due to their low refractive index they are not appropriate as potential embedding media for specimens cleared with BABB or DBE. Anhydride-based curing agents for epoxies have been used in electron microscopy [11]–[13]. However, since they require curing temperatures beyond 60°C, which may inactivate GFP fluorescence, we considered them as less suitable for resin embedding of GFP-expressing cleared samples. Thus we focused our screening for an optimal curing agent on the chemical group of cyclic or non-cyclic aliphatic polyamines.

The resin formulation described here may have various applications besides ultramicroscopy of large samples. Other transparent histological specimens as cell cultures or slices may be embedded for microscopy utilizing high illumination intensities and for long-term storage.
